# Integrating depression care within NCD provision in Bangladesh and Pakistan: a qualitative study

**DOI:** 10.1186/s13033-020-00399-y

**Published:** 2020-08-11

**Authors:** Jerome Wright, Papiya Mazumdar, Deepa Barua, Silwa Lina, Humaira Bibi, Ateeqa Kanwal, Faiza Mujeeb, Qirat Naz, Rahim Safi, Baha Ul Haq, Rusham Zahra Rana, Papreen Nahar, Hannah Jennings, Siham Sikander, Rumana Huque, Asad Nizami, Cath Jackson, the NIHR Global Health Research Group, IMPACT

**Affiliations:** 1 Department of Health Sciences, University of York, Heslington, York, YO10 5DD UK; 2 ARK Foundation, House No 6, Road No 109, Gulshan 2, Dhaka, Bangladesh; 3 Institute of Psychiatry, Rawalpindi Medical University, Rawalpindi, 46000 Pakistan; 4 Brighton and Sussex Medical School, Medical Research Building, University of Sussex, Falmer, Brighton, BN1 9PX UK; 5 Health Services Academy, Islamabad, PM Health Complex, Chak Shahzad, Islamabad, 44000 Pakistan; 6 Valid Research Ltd, Suite 19, Sandown House, Sandbeck Way, Wetherby, LS22 7DN UK

**Keywords:** Non-communicable disease, Depression, Behavioural activation, South Asia, NCD facilities, Mental health policy, Mental-physical co-morbidity, Depression care integration

## Abstract

**Background:**

Co-morbidity of depression with other non-communicable diseases (NCDs) worsens clinical outcomes for both conditions. Low- and middle-income countries need to strengthen mechanisms for detection and management of co-morbid depression within NCDs. The Behavioural Activation for Comorbid Depression in Non-communicable Disease (BEACON) study explored the acceptability and feasibility of integrating a brief depression intervention (behavioural activation, BA) into NCD services in healthcare facilities in Bangladesh and Pakistan.

**Methods:**

Face-to-face qualitative interviews were conducted with 43 patients and 18 health workers attending or working in NCD centres in four healthcare facilities in Bangladesh and Pakistan, and with three policy makers in each country. The interviews addressed four research questions (1) how NCD care is delivered, (2) how NCD patients experience distress, (3) how depression care is integrated within NCD provision, and (4) the challenges and opportunities for integrating a brief depression intervention into usual NCD care. The data were analysed using framework analysis, organised by capability, opportunity and motivation factors, cross-synthesised across countries and participant groups.

**Results:**

Patients and health workers described NCD centres as crowded and time pressured, with waiting times as long as five hours, and consultation times as short as five minutes; resulting in some patient frustration. They did not perceive direct links between their distress and their NCD conditions, instead describing worries about family and finance including affordability of NCD services. Health worker and policy maker accounts suggested these NCD centres lacked preparedness for treating depression in the absence of specific guidelines, standard screening tools, recording systems or training. Barriers and drivers to integrating a brief depression intervention reflected capability, opportunity and motivation factors for all participant groups. While generally valuing the purpose, significant challenges included the busy hospital environment, skill deficits and different conceptions of depression.

**Conclusions:**

Given current resource constraints and priorities, integrating a brief psychological intervention at these NCD centres appears premature. An opportune first step calls for responding to patients’ expressed concerns on service gaps in provisioning steady and affordable NCD care. Acknowledging differences of conceptions of depression and strengthening psychologically informed NCD care will in turn be required before the introduction of a specific psychological intervention such as BA.

## Background

Each year, approximately 41 million people die from non-communicable diseases (NCDs), 71% of all deaths of which over 85% occur in low and middle-income countries (LMIC) [1, 2]. The four main types of NCDs; cardiovascular diseases, diabetes, chronic respiratory diseases and cancer, also represent the major source of global morbidity accounting for three out of every four years lived with disability [2]. The global prevalence of NCDs is increasing rapidly, with a particularly acute rise in South Asia [3–5].

Since 2010, following the roll-out of the World Health Organization Package of Essential Noncommunicable Disease Interventions [6], NCD care facilities have become an important health resource for both the screening and ongoing care of people with NCDs [7, 8].

The burden of physical illness brings with it an associated increased risk of mental disorders [9], with not only shared biological and environmental determinants, but the impact of adjusting to the diagnosis of an NCD, living with pain, disability and social and economic consequences [10]. The prevalence of depression is two to three times higher in people with an NCD [11, 12]. A recent systematic review of hospital-based studies in South Asia, revealed a pooled estimate of depression of 40% in patients with diabetes, 37% for patients with cancer, 38% in patients with hypertension, 39% in patients with stroke, and 44% in patients with chronic obstructive pulmonary disease [13]. This co-morbidity of depression with an NCD is important because it worsens the outcomes for both and is associated with poorer self-management and treatment adherence, reduced treatment response and higher morbidity and mortality for both [14, 15].

Despite this burden and calls for developments in practice [9, 16], the detection and management of depression in NCD care remains a major challenge particularly for LMICs [14]. While research evidence and clinical guidelines indicate that depression is amenable to treatment with relatively low cost psychological and pharmacological therapies [17], the acceptability, feasibility and effectiveness of such approaches integrated and delivered at NCD care facilities is unknown. Policy initiatives in both Pakistan and Bangladesh have recognised and designed potential responses to the challenge of mental and physical co-morbidity and were included in one foresighted national plan in Pakistan [18]. Despite this, as in other settings, resource-stretched facilities continue to prioritise their central mission of responding to physical health needs and rarely offer defined and standardised treatments for depression [19].

With this in mind, the Behavioural Activation for Comorbid Depression in Non-communicable Disease (BEACON) study set out to examine the acceptability and feasibility of integrating an existing brief depression intervention (behavioural activation, BA), into NCD centres of Bangladesh and Pakistan. BA is a brief psychological intervention highlighting the role of positive reinforcement in behaviour change [20]. In accordance with recommended practice for adapting health interventions [21], to specifically adapt BA to the context and population it will be targeting: in this case, four designated NCD centres in two districts across each of Bangladesh and Pakistan. Our first step was to thoroughly assess target communities by undertaking surveys of organisational capacity at each NCD centre and the national clinical and policy environment context. We then conducted qualitative interviews with key stakeholders, including patients and health workers across the NCD centres and mental health/NCD policy makers from each locality. This paper reports on these in-depth interviews to investigate:

1. How NCD care is delivered at the NCD centres,
2. Whether and how NCD patients experience mental distress,
3. Whether and how depression care is currently integrated within care at the NCD centres, and
4. The challenges and opportunities for integrating a brief depression intervention at these NCD centres.

## Methods

This was a qualitative interview study conducted between July 2019 and March 2020.

The theoretical framework underpinning the study was the Capability-Opportunity-Motivation-Behaviour (COM-B) model that identifies the inter-linked factors of capability (psychological, physical), opportunity (social, physical) and motivation (reflective, automatic) as influencing behaviour [22]. Details on these factors are presented in the findings.

### Setting

In both countries NCD care is delivered through a three-tier healthcare infrastructure. Primary and secondary care facilities are mandated to provide screening services, basic diagnoses, treatments and refer to tertiary facilities as required. The research was conducted at the above mentioned NCD centres: a primary and a secondary healthcare facility in Narayanganj district, Bangladesh, and a secondary and a tertiary care facility in Rawalpindi district, Pakistan.

### Recruitment and participants

The situational analysis conducted prior to the interviews, provided information on staff groups who might potentially deliver a brief intervention for depression. Based on this, within each NCD centre we planned to interview five health workers (including one health manager) and 12 patients (ensuring a mix of men and women withcardiovascular disease, diabetes and respiratory disease). Whilst cancer is identified as one of the four main types of NCD [2], the situational analysis revealed that the treatment and management of cancers is undertaken at general medical outpatient or specialised cancer units rather than NCD centres.

Patients and health workers were approached at the NCD centres and those who agreed were interviewed. Very few declined, just one health worker in Bangladesh and 3 patients in Pakistan. Forty-three NCD patients were interviewed (24 Bangladesh; 19 Pakistan). They were a mix of men and women (50% male in Bangladesh; 58% male in Pakistan), aged 30–70 years in Bangladesh, 27–67 years in Pakistan. Just over half (58% in both countries) had received no education or only completed primary education. Patients had a range of NCDs (one third with each in Bangladesh; diabetes 42%, CVD 32%, respiratory disease 26% in Pakistan).

Eighteen health workers were interviewed (eight health workers, two health managers in Bangladesh; six health workers, two health managers in Pakistan), a mix of men and women (40% male in Bangladesh; 50% male in Pakistan). In Bangladesh six were nurses, four were doctors and they all treated patients presenting with NCDs. In Pakistan, all but one were doctors including specialists and general practitioners. The other participant was a senior IT administrator working on the reception desk.

Three policy makers with a remit for mental health and/or NCD provision in each country were identified using existing networks and contacted by telephone or email to recruit to the study. One policy maker declined in Bangladesh. Two thirds were men (in both countries). In Bangladesh, the participants had a remit for primary care, mental health and NCD care respectively. In Pakistan, two participants worked on NCD policy/programmes and the other specialised in mental health.

### Data collection

In-depth interviews were conducted by the research teams (DB, SL in Bangladesh; HB, AK, QN RZR, RS in Pakistan). They had a mix of experience of qualitative research methods and received virtual training from CJ with a colleague from the University of York. Interviews were conducted in the local language at the NCD centre or the policy maker's place of work. They were later transcribed verbatim, anonymised and all checked for accuracy against the audio-recording by the researcher who conducted the interview. One quarter was translated into English. This enabled us to retain the meaning and context captured in the local languages for most interviews, whilst permitting cross-country team working and supervision in English.

The situational analysis provided an overview of the systems and processes within each NCD centre. To build on this insight and informed by COM-B [22] the interview topics discussed with patients their experiences of living with an NCD, of receiving NCD healthcare, and their views on integrating mental healthcare in the NCD centre. Interviews with health workers explored their experiences of delivering NCD care and depression management, and their views on the challenges and opportunities to integrating a brief depression intervention into their NCD service provision. Finally, interviews with policy makers focused on current NCD, mental health and combined policies, and the potential for integrating a brief depression intervention into NCD service provision. The topic guides were piloted to streamline and improve clarity, apart from the policy makers’ due to a lack of potential participants.

The duration of patient interviews ranged from 26 to 73 min (some required additional explanation of the questions); 38 to 76 min for health workers. Interviews with policy makers were slightly longer (60–90 min).

### Data analysis

The data were then subjected to thematic analysis using the Framework approach [23] which is designed to address programme or policy-related questions. We used a mix of deductive and inductive approaches [23]. CJ provided training and detailed feedback on each step of the process. The data analysis team were DB, SL (Bangladesh); HB, BUH, AK, FM, QN, RZR, RS (Pakistan) and CJ, HJ, PM, PN, JW (UK).

#### Within-country analysis

An English language thematic framework was developed for each participant group based on the study research questions, topic guides, COM-B [22] and two interview transcripts. The draft frameworks were piloted with another 1–2 transcripts per country, before finalising. The thematic frameworks were then systematically applied to the interview data. Summaries of responses from participants and verbatim quotes were entered. These charted data were reviewed and interrogated to compare and contrast views, seek patterns, connections and explanations. Descriptive findings were written for each participant group in each country, focusing specifically on addressing the four research questions and drawing on the principles of thematic analysis [24].

#### Cross-community synthesis

The final step was a thematic cross-community synthesis that took account of the inferences derived from all the interview data. Using the Descriptive Findings documents, the data across both countries were reviewed to explore similarities and differences in views across and within healthcare facilities and countries, and for different participant groups (patients, health workers, policy makers). We also looked for gender and NCD patterns within the patient data.

## Findings

### Participants’ views and experiences

Participants’ accounts are organised by the research questions. Some were more relevant to particular participant groups, evident from the data presented. Where there are differences by country, healthcare facility, participant group, NCD or gender these are highlighted.

#### Q1. How is NCD care delivered?

All four NCD centres provided the same services: screening and monitoring (e.g. blood pressure, blood glucose, height/weight), referral for diagnostic tests (e.g. ophthalmic tests for diabetes patients), advising on self-management (e.g. lifestyle, taking medication, attending follow-up appointments) and issuing prescriptions for medications.

“New” and existing NCD patients attended the services, some who had received treatment for many years, 14 years for one diabetes patient. Patients self-referred and could access this care, free of charge. Health workers described patients presenting with physical symptoms related to their NCD, for example breathlessness (respiratory and cardiovascular disease), chest pain and headaches (cardiovascular disease), high blood sugar, foot pain and fatigue (diabetes). They also mentioned that patients come with psychological and social issues related to their NCD (see below, Q2).

Despite Bangladesh and Pakistan both having relatively well-structured health systems, policy makers observed that they struggle to provide efficient NCD services. On the frontline, it was clear from patients and health workers that these NCD centres were incredibly busy, crowded environments. Health workers in Bangladesh reported treating 100 to 150 patients a day in the primary healthcare facility, of which 10 to 15 had an NCD, and 50 to 60 patients a day in the secondary healthcare facility, 10 with an NCD. In Pakistan, the numbers were typically 50–60 NCD patients a day in the tertiary facility and 100–200 in the secondary facility. This high volume meant there could be long waiting times (reported by patients as up to 3 h in Bangladesh and 5 h in Pakistan) in crowded rooms often with insufficient seating.

"When a person comes, he has to wait in line at four places and his whole day gets wasted. First, we make a chit [appointment slip], then we get our blood pressure checked, then blood sugar level, and then they give us prescription chit. Then they prescribe medicine and then we get in line at the medical store."

(P_A_PM08: male patient with diabetes, tertiary care facility, Pakistan).

The busy clinics also meant that appointments could be as short as five minutes with the doctor. Health workers and patients both recognised this was insufficient. Some patients considered that their health concerns were explored in detail, having received advice on related lifestyle issues e.g. nutrition, physical activity. Others were less satisfied, complaining that health workers did not listen, focused only on physical assessment, prescribing medication and asserting the importance of concordance.

“Due to time constraints it is tough to counsel patients like about the consequences of not taking medicine regularly, and the side-effects of the medicines.”

(B_S_HW45: consultant doctor, primary care facility, Bangladesh).

"Doctors just said that you have to take your medicines with caution, do not miss any dose, need to take all diabetes medicines and take care of yourself."

(P_A_PF24: female patient with cardiovascular disease, tertiary care facility, Pakistan).

A further implication of the busy clinics was a strain on medication supply. In Bangladesh, whilst most patients had received free medication for cheaper medicines e.g. antihypertensives, nebulisers; a few had been asked to pay for more expensive medicines like insulin. A lack of availability of medicines in Pakistan meant some patients had to collect and pay for them at a private pharmacy. These medication costs were recognised by health workers and patients as prohibitive for some, potentially leading to poor adherence to medicine regimes.

“If medicines are prescribed from here, I collect them from the pharmacies outside. Here, the cheap medicines are available only – paracetamol, anti-ulcerants, anti-hypertensive drugs, drugs for fever. They do not provide medicines for diabetes, so I buy it.”

(B_S_PF29: female patient with diabetes, primary care facility, Bangladesh).

"Whenever we come to get insulin, they tell us there is no insulin, insulin is provided on a separate day and sometimes when we come on that day, they say that insulin is finished. Then we don’t take it because I am a poor man and I cannot afford it."

(P_A_PM08: male patient with diabetes, tertiary care facility, Pakistan).

Despite these frustrations, patients continued to attend the NCD centres because they were accessible (free of charge), and the majority view was that doctors were competent and delivered good care. Indeed, patients’ overall assessment of their care appeared to be based on its effectiveness. In other words, it was viewed as “good” when NCD symptoms were reduced and poor when health did not improve.

“I’m fine with the treatment that I am getting because when I came here, my condition was worse and now I’m able to talk and can walk to the washroom by myself. I follow all the advice.”

(P_A_PM09: male patient with cardiovascular disease, tertiary care facility, Pakistan).

#### Q2. Do NCD patients experience distress—how?

Patients frequently described cognitive (negative thoughts, pessimism), affective (low mood, anger, worry, irritation, feeling stressed) and somatic (tiredness, headache, insomnia) symptoms commonly accepted as depression. Health workers also mentioned patients presenting with these symptoms.

“Sometimes I do not feel like doing anything, not even eating a meal. Sometimes I feel like crying, from sorrow. Because I cannot take my medicines properly, cannot do anything properly.”

(B_N_PF26: female patient with respiratory disease, secondary care facility, Bangladesh).

“Patients have insomnia, they share their emotions, saying I sit all night and I was feeling panic and have been fighting a lot or weeping.”

(P_A_HW42: house officer, secondary care facility, Pakistan).

Health workers in both Pakistan facilities estimated that 50% of NCD patients presented with mental health issues including depression, with one health worker in the secondary care facility suggesting it was as high as 90%. In Bangladesh, these estimates were 50% (primary care) and 20–60% (secondary care). In both countries, their perception was that the typical profile of these patients were women. A few health workers in Bangladesh believed that people with no/low education suffered more from depression as they were less likely to be able to accept their NCD diagnosis. Three health workers in Pakistan mentioned education, oscillating between either being highly educated or not educated as associated with depression.

Health workers’ interpretation and patients’ accounts suggested that patients did not typically view symptoms as a “separate illness” to their NCD or use the term “depression”.

“Our patients are not oriented to it [mental health]. They are in depression, but they do not understand it. They are saying that they are suffering from diabetes or hypertension but the depression is a burden for the patient. It is a greater mental trauma than their diabetes, but patients do not have that understanding. They think that every disease is about physical illness.”

(B_N_HW44: Medical officer, secondary care facility, Bangladesh).

Instead, distress was largely associated with their NCD and patients’ links to physical symptoms and disability as well as anxiety about the disease itself and the prognosis. Some patients referred back to when they were diagnosed, recalling their worry at that time.

“Most of the patients are suffering from depression because they think that these disorders are chronic illnesses and are only controllable but not curable and somatization of their symptoms.” (P_B_HW43: medical officer, tertiary care facility, Pakistan).

“I had a lot of adjustments during first three months of being detected with high pressure. I was quite devastated mentally. I was scared because, if I die with stroke, what will happen to my family?”.

(B_N_PM01: male patient with cardiovascular disease, secondary care facility, Bangladesh).

Interconnected to patients’ worry about their NCD and the reported causes of distress were concerns about the costs of treatment, implications of their illness for work, strain on family life and what should happen if they die. Women commonly related these worries and distress to family, whilst men spoke more about money concerns, including providing for their family.

“I keep on thinking that even whilst I can earn today, I am unable to pay for 2000 BDT to purchase medicines. There will be a time when I will not have any means. I won’t be able to earn my living?”.

(B_N_PM03: male patient with diabetes, secondary care facility, Bangladesh).

It was unclear from the interview data whether this distress was ascribed as an actual symptom of their NCD. Patients with diabetes tended to directly link their tiredness and headaches to their physical health. Irrespective of these attributions, it was clear that for patients only a reduction in NCD symptoms or complications would reduce their distress.

Non-NCD related causes of symptoms of depression were also identified by patients and health workers. These typically focussed on (un)employment, financial and family stresses (e.g. divorce, poor relations with in-laws, difficulties with children). A small minority were adamant that their psychological health was completely unrelated to their NCD.

“Poverty is one cause of depression in Pakistan.”

(P_A_HW42: house officer, secondary care facility, Pakistan).

“I feel stress because of my children, I am tired and worried. This is not related to the disease.”

(P_B_PF21: female patient with diabetes, tertiary care facility, Pakistan).

Finally, a few health workers in both countries spoke of the negative impact of depression on patients’ self-management of their NCD, seen to threaten good medication compliance and general self-care.

“They do not come for follow-up. That means they become tired and think not to take medicine anymore. They may come again to the facility after two or three years; meanwhile their health has deteriorated.”

(B_N_HW44: Medical officer, secondary care facility, Bangladesh).

#### Q3. How is depression care integrated within NCD provision?

A strong message from health workers and policy makers was that there are currently no specific guidelines or training for treating depression, and no standard screening tools or system for recording patients’ depression diagnosis or care at the NCD centres.

Without exception, health workers across all four NCD centres used intuition to identify depression in patients; from their behaviour, for example crying, subdued, not following self-management advice; their untidy appearance and negative body language; and the cognitive, affective and somatic symptoms that patients describe (outlined above, see Q2). In Pakistan they spoke of using a “deductive method” during assessment concluding that a patient may be depressed if the symptoms they describe were not associated with their NCD. They also relied on family members’ feedback to assess depression.

”The depressed patient’s face tells that they are actually suffering from depression. They smile less, and seeing their face it can be understood that they have lots of sorrow in their mind. These patients do not always express their mental pain.”

(B_S_HW45: consultant doctor, primary care facility, Bangladesh).

The absence of guidelines was seen to run the risk of health workers not asking the right questions and a patient’s depression being missed.

“There is no proper checklist for diagnosis and treatment system of depression, so there are chances that patients might get missed.”

(P_B_HM61: consultant doctor, tertiary care facility, Pakistan).

Another perceived challenge was that patients may choose to not share with health workers how they are feeling in terms of their mental health. Indeed, a stigma associated with having depression was mentioned by health workers and policy makers. In Pakistan, they also wondered if patients would see discussing mental health as a legitimate remit of the NCD doctor.

”They think ‘If I express such things then the society will be little me and will address me as a lunatic’. Though this [depression] is a very normal thing and has treatment, but they do not want to disclose it.”

(B_N_HW44: Medical officer, secondary care facility, Bangladesh).

Health workers spoke of delivering two different treatments for depression—anti-depressant medication and ‘counselling’; severe cases were referred to psychiatry. The pressure of time meant that while prescription of medication was straightforward, there was insufficient time for counselling. Value was placed on interacting with patients in a way that ‘consoled’ and ‘motivated’. It was clear that these conversations focused on NCD management not mental health, because this was the health workers’ area of expertise.

“If we think they have depression then we start them on antidepressants. We treat them pharmacologically. Usually these kinds of patients are referred to me for treatment by the NCD doctor.“

(P_B_HM61: HM61: consultant doctor, tertiary care facility, Pakistan).

In addition to having no formal guidelines for assessment or treatment, there were also no formal recording mechanisms for patients’ depression diagnosis or treatment. Only NCD details of patients were recorded.

”We do not have a register for mental health patients. There are two register books, one is for general patients and another one is for NCD patients.”

(B_N_HW43: senior staff nurse, secondary care facility, Bangladesh).

Health workers were clear they had not been formally trained in mental health, except doctors who had done a rotation in psychiatry during their medical training. In Bangladesh doctors with this background were perceived to be better at recognising patients’ distress as depression, and they appeared in their interviews to be more familiar with mental health protocols used elsewhere. Whilst experienced NCD doctors in Pakistan were considered as able draw on their general medical experience, junior doctors clearly could not. Overall, NCD doctors lacked confidence in this field and formal training was seen as required.

“A doctor sitting in the NCD corner does not have enough knowledge and skills relating to psychiatry and mental health.”

(P_B_HM61: consultant doctor, tertiary care facility, Pakistan).

#### Q4. What are the challenges and opportunities for integrating a brief depression intervention into NCD provision?

All three participant groups were presented with a description of a brief depression intervention (BA) in their interview and asked their thoughts on its potential integration into NCD care. The opportunities and challenges they identified are presented here, organised by the COM factors [22].

##### Capability (psychological, physical)

Capability presented as a challenge related to the lack of relevant knowledge and skills of health workers and low levels of understanding about mental health amongst patients.

As described above (see Q3), health workers in both countries acknowledged their lack of training, confidence and expertise in depression care. Indeed, there was some misunderstanding that BA requires mental health specialists to deliver it. They were clear that to deliver this brief intervention they would need training on the rationale, content and delivery mechanism of this “talking therapy”. They were motivated to receive this training (see *Motivation)* and once trained thought they would be competent to deliver BA.

Policy makers also recognised the lack of health workers trained in mental health, a particular challenge for Pakistan. In Bangladesh, they reported national commitment to train all health workers in mental health and NCDs. However, the scale and pace of training were lagging far behind the targeted levels and there was perceived to still be a “mental health gap” in service provision.

“For non-communicable diseases and mental health they [health workers] don’t have the skilled people, this requires training and capacity building.”

(P_PM72: policy maker with remit for NCDs, Pakistan).

Health workers and policy makers suggested that a potential implication of poor awareness and understanding of depression amongst patients (described above, see Q2), particularly those with low levels of education, was that they may not perceive a need to attend for mental health support or be aware it is available. In Pakistan several health workers and a policy maker believed that patients come to the NCD centres wanting medication and it will require a huge cultural shift to propagate knowledge of the use of psychological and behavioural science principles instead of pharmacological treatments in the general healthcare of NCD patients.

“Patients are okay with medication, drugs, and access to treatment issues, but to convince them of the importance and the role of non-pharmacological interventions is another policy challenge."

(P_PM73: policy maker with remit for mental health, Pakistan).

Patients’ own views were that they had never heard of this type of talking therapy and several would want more information about it before signing up to participate. Indeed, within the interviews they struggled to understand what the BA intervention was from the brief description provided. It was clearly misunderstood to be an opportunity to discuss their NCD with a health worker (described further in *Motivation*).

"It should focus on medicine and to ensure regular check-ups and facilitation. It should decrease our fatigue. That way we will feel less stressed and that would be great."

(P_A_PM08: male patient with diabetes, secondary care facility, Pakistan).

##### Social opportunity

In terms of social opportunity, stigma associated with depression was perceived by health workers as a barrier to patients attending for this type of treatment in Bangladesh (described above, see Q3). Social support for patients attending for additional appointments was another factor, particularly for women in Bangladesh. For the health workers, a significant challenge was a combination of their very busy roles in delivering NCD services (described above, see Q1, Q3).

There were mixed views amongst patients about the support they received from their family and friends to attend the NCD centre, and whether this support would be available for them to come for a psychological treatment. In Pakistan men and women usually reported that they were not accompanied to the facility by others and didn’t necessarily receive help with household/childcare responsibilities to attend. Whereas in Bangladesh, several participants reported that people would come to the NCD facility with them. Here, challenges for women were particularly evident, both in terms of needing support with household/childcare duties and requiring their husband’s permission to attend. One woman mentioned several times in her interview that she had a disabled son and no one to help look after him so her husband would not allow her to attend the facility.

“He [my husband] is the primary guardian, I have to inform him that I am going to such-and-such place today, or for such-and-such purpose. If he does not permit me to come, how can I come?”

(B_N_PF26: female patient with respiratory disease, secondary care facility, Bangladesh).

##### Physical opportunity

Several physical opportunity challenges, and some drivers, for integrating the brief depression intervention into NCD care were evident. These related to health worker and patient time, physical space in the clinic, patient travel, and the policy context.

The frenzied NCD centre environment and short consultation times (described above, see Q1) meant that health workers were unable to see how it would be possible to include a brief depression intervention within their NCD duties, unless they were scheduled to focus solely on that. Some identified a need for additional “nominated” staff and a dedicated space.

“They [doctors] in a whole day 2–3 min are given to patients for examination. BA sessions are not possible along with line of duty in OPD [outpatient department]. If 250 patients come there and 100 of them are associated with symptoms of depression, how can they adjust 100 patients each day for 45 min per patients for session.”

(P_A_HM62: assistant professor of medicine, secondary care facility, Pakistan).

“Yes, there is a lack of manpower. Plus, the room is small. If I talk to a patient, another is listening. I think that if there is a separate room then the doctor can check him and can understand whether the patient needs behaviour therapy [BA] or not, then s/he can send the patient in the separate room to receive it. But it is not possible to do it in the same room.

(B_N_HW43: senior staff nurse, secondary care facility, Bangladesh).

Time was also mentioned by patients; specifically, time needed to travel to the health facility (ranging from 15 min to 7 h) and time spent in the waiting room (described above, see Q1). Most said they could find time to attend, although some would need to organise appointments to align with family and work commitments.

“There are many garment workers. They cannot make time other than Friday. If you call her/him and say, “Today is Sunday [a working day], please come to the centre and provide us some of yours time” can s/he provide it? No, s/he cannot.”

(B_N_PM03: male patient with diabetes, secondary care facility, Bangladesh).

“I travel for my NCD and I face problems so I cannot come for it due to traveling issues, I travel 2 to 3 h to reach the hospital.”

(P_A_PF05: female patient with diabetes, secondary care facility, Pakistan).

A further travel-related issue for patients was the cost of public transport. Whilst men in Pakistan often had their own transport, most patients travelled to the health facility by rickshaw, bus, vans or autos, often for long journeys (a 20 km journey mentioned in Bangladesh, up to 3 h’ travel mentioned in Pakistan). Approximately one third of Bangladeshi patients and nearly half of the Pakistani patients mentioned this financial cost of coming to the NCD facility, recognising that for some, this was prohibitive.

“I do not have the money to come here every week, and obviously I don’t have ability to come here daily. I cannot manage, it is very difficult for me.”

(B_S_PF32: female patient with cardiovascular disease, primary care facility, Bangladesh).

In summary, an implicit message from patients was that the BA appointments need to be co-ordinated with their existing NCD visits; to reduce the impact of time and cost requirements; or otherwise additional travel expenses should be reimbursed.

The readiness of the policy context to support integration of a brief psychological intervention into NCD centres was different in each country. In Pakistan, whilst mental health was a priority, policy makers believed that government level commitment and associated funding was lacking for prioritising mental health within NCD care.

“To try and convince people who were not ready to bring mental health issues into the fold of non-communicable diseases—this was a huge challenge. The attitude of people was that mental health is not a part of the grand scheme of things.”

(P_PM73: policy maker with remit for mental health, Pakistan).

On the other hand, the commitment for integrating mental health and NCD care in Bangladesh, was evident within the Mental Health Act and associated financing. Multiple NCD initiatives were underway. Their challenges were a lack of national level data to inform initiatives and delays in decision-making due to new jurisdictions within directorates.

##### Motivation (reflective, automatic)

Health worker and patient motivation for the integration of a brief depression intervention was generally positive (albeit based on some misunderstanding of what it entails). However, it was clear that the capability and opportunity challenges described above would be potential threats to these positive intentions becoming reality. In addition, patients' prior experience of their NCD care emerged as an important factor in their motivation.

Health workers believed in the value of a talking therapy for treating NCD patients with depression. They would be motivated to take on this work (seen as additional to their existing responsibilities) if it improves patients’ recovery, or if they personally gain new knowledge through formal training, and are rewarded by financial incentives or management appreciation.

“Recovery of patients is a motivation for them. They [health workers] will feel proud of themselves and their seniors as well for recovery of patients.”

(P_B_HW44: trainee of vocational training institute, tertiary care facility, Pakistan).

“If there is provision of some incentives, it would be better. Willingness is the main thing. Without it, nothing can be done.”

(B_N_HM61: resident medical officer, secondary care facility, Bangladesh).

Patients had clear enthusiasm for this brief depression intervention. However, this was based on a misunderstanding about what this intervention is (described above, see *Capability*). In reality, they were keen to have the opportunity to talk to a health worker about their NCD as there was no time for this within their consultation. Most appeared particularly motivated by the idea of improving their NCD rather than their mental health; therefore, if BA offered the potential for this, they were keen to try it.

"This programme is good. We can change our mind through it and people will automatically get better."

(P_B_PM12: male patient with respiratory disease, tertiary care facility, Pakistan).

“Patients always expect to have treatment that provide cure.”

(B_N_PF26: female patient with respiratory disease, secondary care facility, Bangladesh).

As a slight caveat to this enthusiasm, particularly amongst men in Pakistan, motivation appeared to be influenced by patients’ experience of NCD treatment; namely a poor experience, for example, insufficient or rude doctors, long waiting times and medication not always being available (see Q1) threatened their willingness to come back for this type of programme. Conversely a kind, helpful doctor, and a nice setting would motivate them to attend.

"Just that everything should be good, the doctors should be good, the poor people should get assistance. The person becomes happy. Once the patient doesn’t speak the disease gets worse. I believe that the doctor should speak to patients in a good manner. It’s why the patient comes to the doctor to get consultation."

(P_A_PF23: female patient with respiratory disease, secondary care facility, Pakistan).

In Bangladesh, the patients spoke less of the impact of a negative experience, focusing instead on the positive features they would like—a pleasant physical environment (e.g. nice chairs in the waiting room, a private room for consultations) and certain attributes of the person delivering the BA intervention (e.g. kind, well-educated, credible).

”A separate room should be used for conducting behavioural activation. No queue of patients should be here. Patients should not feel uneasy. There should be seating for doctors and patients, there should be fans for cooling.”

(B_S_PF27: female patient with cardiovascular disease, primary care facility, Bangladesh).

## Discussion

With the growing appreciation of the impact of depression on the lives of people with NCDs and the accompanying calls to integrate depression treatment within NCD care across the globe [14] there has never been a more important time to establish acceptable, feasible and effective ways of achieving this. By grounding such aspirations within the reality of NCD care provision in Bangladesh and Pakistan and through undertaking a thorough assessment of the target communities [21], this study has revealed important socio-cultural contextual findings that show that the challenge to integrate depression care does not rest with resource limitation alone. An analysis of the factors that promote change using the COM-B framework [22] has uncovered a number of potential inter-linked drivers and critical barriers, relating to capability, opportunity and motivation, to delivering a psychological intervention and a crucial gap between demand and supply perspectives.

The opportunity to deliver a discrete psychological intervention within the current NCD centre environments across the four healthcare facilities was extremely limited. However, health workers and policy makers across the countries were interested and motivated to improve depression management. This could be tapped as a potential driver to integration of depression care, supplemented with a significant increase in human and physical resources. Health workers at each NCD centre were aware of current constraints for managing depression, including limited availability of anti-depressant medication and the time-constrained consultations where ‘counselling’ could only be offered within brief consultations for advice on treatment and lifestyle change for the NCD. Health workers’ capability of delivering an intervention such as BA would need to start with adopting a more standardised approach to assessing depression, replacing the current ‘deductive’ method. Although again, the opportunity cost of workers delivering psychological interventions as opposed to current duties remains a substantial consideration.

Significantly, the study findings showed that while the vast majority of patients associated the experience of distress with having the NCD, they saw the source of this as less of a difficulty with their own mental adjustment to living with the NCD than as a result of i) the impact of physical illness on their ability to function and to fulfil their roles in life (family, social and economic impacts) and ii) pre-existing problems of poverty that exacerbate their difficulties, (lack of resources to travel, to afford investigations, the fluctuating availability of medication and inability to afford to buy this privately).

These structural deficits were the primary concerns of patients and contributed significantly to their levels of uncertainty and unpredictability which increased anxiety and hopelessness. Although the topics of concern differed according to gender roles (e.g. responsibilities for childcare or earning wages for the family) these social attributions of distress did not differ between genders.

The prominence of social factors as the cause of depression has been reflected globally [25, 26], in people with NCDs [27] and in similar investigations in Bangladesh [28, 29] and Pakistan [30]. The experience of co-morbidities adds a particular focus on need for this population [15]. For solutions to their distress, patients in this study looked towards practical change in care provision rather than valuing emotional-focused treatments [31]. They perceived their concerns would be mitigated by improved NCD care and access to treatments. The patients recognised benefiting from having more time to discuss their concerns with health workers. However, since they had not previously considered that they were ‘depressed’, they were not sure if such discussion would take the form of a standardised psychological intervention. Where psychological distress was acknowledged by patients, similar to their NCD care, patients were orientated towards physical treatments (medication) over a talking therapy [32]. Patients were also wary of the stigma that would attach to any suggestion that they were mentally—as well as physically—ill.

This gap in shared understanding of the ‘problem’ facing patients represents a major barrier to patient acceptability and the demand for a psychological intervention. Differing expectations and demands are directly linked to explanatory models of distress [33, 34]. Clearly concerns about the impact of a physical illness on a person’s life is a familiar but not universal way to frame distress. What health workers described as patient’s lack of understanding’ of depression, simply reflects explanatory models and culturally embedded understandings of distress that are different but equally as socially constructed as biomedical understandings [35]. For any therapeutic exchange, aligning perspectives of patients and providers is a vital first step since it creates the positive expectancy that increases positive outcomes [36]. This is made more complex with pluralistic socio-cultural differences of conceptualising problems and identifying solutions [37]. It is possible for patients to be provided ‘depression- literacy’ so that they frame the problem in this way and become orientated to a specific model of treatment [38] and many such interventions elicit high effect-sizes compared to ‘usual treatment’ [39]. However, in this regard, the study also noted a concern when introducing the potential of a BA—informed intervention within the interviews with participants that highlighted both differing cultural conceptualisations of depression [40] and the additional complication of physical illness when integrating BA into the care of people with co-morbidities. All stakeholders in the study equated BA with the lifestyle advice to change behaviour delivered as routine NCD care rather than recognising the specific relationship between activity, positive reinforcement and mood. Whether such a misunderstanding of the behavioural mechanism of BA has been noted in studies where BA has been embedded within other interventions [41] or as a stand-alone approach (e.g. [42].) is unclear. The study has certainly revealed an unfamiliarity with conceptualising depression treatment in this way which perhaps becomes most apparent where the patient population has a concurrent NCD. Overcoming such barriers are complex yet not in themselves insurmountable [43]. However, this reinforces the importance of cultural adaptation, since such differences between training and delivery have been noted in other mhGAP interventions [44].

Nevertheless, aligning attributional and therapeutic perspectives would surely mean listening to the existing explanatory models—not least, patients’ concern for the proximal structural problems in the resources and organisation of NCD care that continually present them with uncertainties. Such a question illustrates an ongoing tension in ‘global mental health’ over the extent to which structural social determinants of distress are submerged by the development of individualised therapeutic interventions [45]. This study indicates a moral legitimacy in first addressing improvements to the provision of appointments, investigations, and the availability of free medication for all patients before focusing exclusively on integrating brief psychological interventions. Nichols’ [46] three central tenets of psychological care: (i) competent physical healthcare, (ii) accurate and accessible information concerning the physical complaint and (iii) appreciation of the psychological impact that such physical health concerns bring [46] appear to offer a useful bridge between structural and individual approaches in the care of people with physical illness. This approach is endorsed in this study by patients’ reflections on their appreciation of positive values and behaviours of health workers. Such principles establish a local awareness of the importance of psychological health and well-being to coping with NCDs. Consideration of strengthening social welfare and support at the NCD centres would in addition provide a practical response to patients’ expressed concerns. Building on this, future approaches such as task-shifting mental health roles to deliver psychological interventions and establishing collaborative care can then potentially drive to design specific psychological interventions integrated into NCD care [16, 47].

It is important to reflect on the strengths and limitations of this study. First, this is a large qualitative study. We interviewed 67 key stakeholders, a mix of patients, health workers and policy makers in two countries. We did not quite achieve our target sample of patients or health workers in Pakistan. This was due to a Dengue fever outbreak that resulted in cardiac and respiratory wings of one hospital to be converted to Dengue care units. In reflecting on whether we achieved generalisability (as a qualitative concept [23]), we have no reason to believe that the study participants would be different to other patients or health workers attending/working in these types of publicly funded NCD centres or other national policy makers. Furthermore, we achieved data saturation (where no new themes were emerging) and captured good diversity of views, providing a valuable breadth of insight. This, and the rigour of the study design and conduct, give us confidence in our findings. A second strength is the use of the COM-B model [22] that provided a comprehensive, theory-informed approach to identify determinants of behaviour and ensured that we considered a broad range of a demand and supply factors. Third, an important part of this work was the research capacity development, specifically in qualitative data collection and analysis methods. A potential limitation is that our brief explanation of BA offered to participants was insufficient for participants to comment meaningfully on its content. However, their feedback on the concept of a brief depression intervention and its delivery was sufficient, alongside our conclusion that integrating this intervention would not be a priority at this time.

## Conclusion

The study has highlighted significant capability, opportunity and motivation barriers, drivers and competing complexities to introducing an approach to depression management in NCD care. While all stakeholders agreed that concerns presenting as depression were present in patients and arose from a combination of physical, psychological, social and economic challenges, it would only be through the allocation of significant additional resources in the form of fully supported implementation systems that BA or an alternative intervention could feasibly be introduced into such NCD centres. In addition, the gap between patient and health worker conceptions of depression and patients’ concerns over their receipt and affordability of NCD care represent a significant barrier in their demand for a psychological intervention. Implementing a discrete intervention of this type would therefore appear premature ahead of attention to optimising patients’ experience of NCD care and aligning more closely the patient and health worker conceptions of distress.

## Data Availability

The datasets used and/or analysed during the current study are available from the corresponding author on reasonable request.

## Abbreviations

COM-B: Capability, opportunity, motivation-behaviour
LMIC: Low and middle income country
NCD: Non-communicable disease
PEN: Package of sssential non-communicable disease interventions
WHO: World Health Organisation
